# Network meta-analysis of the efficacy of nine drugs for cognitive function in patients with Alzheimer's disease

**DOI:** 10.1177/25424823261422205

**Published:** 2026-02-06

**Authors:** Shanshan Huang, Yunyun Guo

**Affiliations:** 1 Department of Life Sciences, 4615 Imperial College London, London, UK; 2 Ageing Epidemiology (AGE) Research Unit, School of Public Health, 4615 Imperial College London, London, UK

**Keywords:** Alzheimer's disease, cognitive outcomes, monoclonal antibodies, network meta-analysis, placebo response

## Abstract

**Background:**

Alzheimer's disease (AD) remains a global challenge, and the comparative cognitive efficacy of emerging pharmacotherapies is still unclear.

**Objective:**

To compare and rank nine pharmacological agents against placebo in AD with respect to key cognitive outcomes using a network meta-analysis.

**Methods:**

We systematically searched randomized controlled trials published up to May 2025 that evaluated aducanumab, lecanemab, donanemab, gosuranemab, semorinemab, tilavonemab, zagotenemab, masupirdine, or sodium oligomannate in AD. The primary outcomes were Clinical Dementia Rating–Sum of Boxes (CDR-SB) and Alzheimer's Disease Assessment Scale–Cognitive Subscale (ADAS-cog); Mini-Mental State Examination (MMSE) was a secondary outcome. Treatments were ranked using the Surface Under the Cumulative Ranking curve (SUCRA).

**Results:**

Fifteen randomized trials encompassing 33 treatment arms were included. No drug demonstrated statistically robust superiority over placebo in primary outcomes. Semorinemab and tilavonemab achieved highest SUCRA rankings, but without significant pairwise advantage. For MMSE, aducanumab showed a modest mean difference versus placebo (1.98, 95% CI 0.03–3.93), though evidence of publication bias reduced confidence.

**Conclusions:**

Current pharmacological treatments do not consistently outperform placebo in AD. Tau-targeted antibodies (semorinemab, tilavonemab) display modest but non-significant promise, whereas aducanumab's apparent benefit is likely confounded by publication bias. Further large, rigorous randomized controlled trials and improved preclinical models are essential.

## Introduction

Alzheimer's disease (AD) is the most common cause of dementia and represents a major global health burden in aging populations. Despite decades of research, effective pharmacological options remain scarce. Current investigational approaches target mechanisms such as tau protein hyperphosphorylation, whereas approved therapies primarily modulate neurotransmission or promote amyloid clearance. Despite these advances, their overall clinical benefits remain modest.^1–32^ In recent years, novel biological agents such as aducanumab,^2^ lecanemab,^3^ and donanemab^1^ have been investigated in late-phase clinical trials. While lecanemab and donanemab have demonstrated relatively consistent efficacy profiles and received regulatory approvals in several regions,^1,3^ aducanumab has generated considerable controversy due to conflicting trial outcomes and regulatory decisions.^2^[Table table1-25424823261422205]

**Table 1. table1-25424823261422205:** Characteristics of included studies.

Study (Author, year)	Agent	Phase	Population	Sample size (N)	Dosage & administration	Follow-up duration	Cognitive outcomes	Overall risk of bias
Budd Haeberlein et al., 2022^2^	Aducanumab	Phase III (ENGAGE & EMERGE trials)	Early Alzheimer's disease (MCI due to AD and mild AD dementia)	∼3285 (ENGAGE n = 1647; EMERGE n = 1638)	Intravenous infusion every 4 weeks; low-dose (3 or 6 mg/kg depending on APOE ε4 carrier status), high-dose (10 mg/kg)	78 weeks (18 months)	CDR-SB (primary), MMSE, ADAS-Cog, ADCS-ADL-MCI	High risk
Chen et al., 2025^8^	Lecanemab	Phase III (Clarity AD, Asian subgroup)	Early Alzheimer's disease (MCI due to AD and mild AD dementia; Asian population)	∼288 (subset of global Clarity AD trial)	10 mg/kg IV infusion every 2 weeks	18 months	CDR-SB (primary), ADAS-cog14, ADCS-MCI-ADL, MMSE	some concerns
Dhadda et al., 2022^9^	Lecanemab	Phase II (Study 201)	Early Alzheimer's disease (MCI due to AD and mild AD dementia)	856 randomized (placebo + lecanemab groups)	IV infusion; 2.5 mg/kg biweekly, 5 mg/kg monthly, 5 mg/kg biweekly, 10 mg/kg monthly, 10 mg/kg biweekly	18 months	CDR-SB, ADAS-cog14, ADCOMS, MMSE	some concerns
van Dyck et al., 2023^3^	Lecanemab	Phase III (Clarity AD)	Early Alzheimer's disease (MCI due to AD and mild AD dementia)	1795 randomized (Lecanemab n = 898; Placebo n = 897)	10 mg/kg IV infusion every 2 weeks	18 months	CDR-SB (primary), ADAS-cog14, ADCOMS, MMSE, ADCS-MCI-ADL	some concerns
Florian et al., 2023^10^	Tilavonemab	Phase II	Early Alzheimer's disease (mild cognitive impairment due to AD and mild AD dementia)	453 randomized (Tilavonemab n = 303; Placebo n = 150)	IV infusion every 4 weeks; doses 1500 mg or 4500 mg	72 weeks (∼18 months)	CDR-SB (primary), ADAS-cog14, MMSE	low risk
Mintun et al., 2021^1^	Donanemab	Phase II (TRAILBLAZER-ALZ)	Early Alzheimer's disease (MCI due to AD and mild AD dementia, amyloid- and tau-positive)	257 randomized (Donanemab n = 131; Placebo n = 126)	IV infusion every 4 weeks; 700 mg for first 3 doses, then 1400 mg thereafter	76 weeks (∼18 months)	iADRS (primary); CDR-SB, MMSE, ADAS-cog13 (secondary)	some concerns
Monteiro et al., 2023^5^	Semorinemab	Phase II (Lauriet)	Mild-to-moderate Alzheimer's disease	272 randomized (Semorinemab n = 136; Placebo n = 136)	IV infusion, 4500 mg every 4 weeks	49 weeks (∼12 months)	ADAS-Cog11 (primary), ADCS-ADL, CDR-SB, MMSE	low risk
Shulman et al., 2023^11^	Gosuranemab	Phase II (TANGO)	Early Alzheimer's disease (MCI due to AD and mild AD dementia)	654 randomized (Placebo n = 163; Gosuranemab groups n = 491 across multiple doses)	IV infusion every 4 weeks; doses 150 mg, 700 mg, 2100 mg, or 7000 mg	78 weeks (∼18 months)	CDR-SB (primary), ADAS-Cog13, MMSE, ADCS-ADL	low risk
Sims et al., 2023^12^	Donanemab	Phase III (TRAILBLAZER-ALZ 2)	Early symptomatic AD (MCI or mild dementia) with amyloid and tau pathology	1736 (Donanemab n = 860; Placebo n = 876)	IV infusion every 4 weeks (700 mg for first 3 doses, then 1400 mg); blinded switch to placebo if amyloid clearance met	76 weeks (approx. 18 months)	iADRS (primary), CDR-SB, ADAS-Cog13, ADCS-iADL, MMSE	some concerns
Swanson et al., 2021^13^	Lecanemab	Phase IIb (proof-of-concept, Study 201 extension)	Early Alzheimer's disease (MCI due to AD and mild AD dementia)	856 randomized	IV infusion; multiple dosing regimens: 2.5 mg/kg biweekly, 5 mg/kg monthly, 5 mg/kg biweekly, 10 mg/kg monthly, 10 mg/kg biweekly	18 months	CDR-SB, ADAS-Cog14, ADCOMS, MMSE	some concerns
Toda et al., 2024^14^	Aducanumab	Phase III (EMERGE & ENGAGE, Japanese subgroup)	Early Alzheimer's disease (MCI due to AD and mild AD dementia; Japanese participants)	164 randomized (ENGAGE n = 83; EMERGE n = 81)	IV infusion every 4 weeks; high-dose 10 mg/kg (with APOE ε4 carrier adjustments)	78 weeks (∼18 months)	CDR-SB (primary), MMSE, ADAS-Cog13, ADCS-ADL-MCI	High risk
Ramakrishna Nirogi et al., 2022^15^	Masupirdine (SUVN-502)	Phase II, proof-of-concept	Patients with moderate Alzheimer's disease, on background therapy with donepezil and memantine	564 randomized (Placebo n = 189; Masupirdine 50 mg n = 190; Masupirdine 100 mg n = 185)	Oral administration, 50 mg or 100 mg once daily, adjunctive to donepezil + memantine	26 weeks double-blind treatment + 4-week washout (total 30 weeks)	Primary: ADAS-Cog11; Secondary: CDR-SB, MMSE, ADCS-ADL, NPI	some concerns
Fleisher et al., 2024^16^	Zagotenemab (LY3303560)	Phase II (PERISCOPE-ALZ)	Early symptomatic AD (MMSE 20–28) with intermediate tau on PET; multicenter in North America & Japan	360 randomized (Placebo vs. 1400 mg vs. 5600 mg)	IV infusion every 4 weeks (Q4W); 1400 mg or 5600 mg	100 weeks double-blind treatment	Primary: iADRS (Bayesian disease-progression model). Secondary: CDR-SB, ADAS-Cog, MMSE, ADCS-iADL; imaging (flortaucipir PET), MRI, plasma NfL/p-tau. (PubMed)	low risk
Wang et al., 2020^17^	Sodium oligomannate (GV-971)	Phase II	Patients with mild-to-moderate Alzheimer's disease, aged 50–85 years, meeting NINCDS-ADRDA criteria	255 randomized (Placebo n = 86; GV-971 600 mg/day n = 85; GV-971 900 mg/day n = 84)	Oral capsules, 600 mg or 900 mg daily, divided into 3 doses	36 weeks double-blind treatment	Primary: ADAS-Cog12; Secondary: CDR-SB, MMSE, ADCS-ADL, NPI	some concerns
Xiao et al., 2021^18^	Sodium oligomannate (GV-971)	Phase III	Patients with mild-to-moderate Alzheimer's disease, aged 50–85 years, meeting NINCDS-ADRDA criteria	816 randomized (Placebo n = 407; GV-971 n = 409)	Oral capsules, 900 mg/day, divided into 3 doses	36 weeks double-blind treatment	Primary: ADAS-Cog12; Secondary: CDR-SB, MMSE, ADCS-ADL, NPI	low risk

Traditional pairwise meta-analyses are limited to direct, head-to-head comparisons and cannot assess the comparative efficacy of multiple competing interventions simultaneously. In contrast, network meta-analysis (NMA) integrates both direct and indirect evidence, enabling a comprehensive comparison and probabilistic treatment ranking of different treatments within a single analytical framework.^4,5^

The present study applies a NMA to assess the comparative efficacy and safety of nine active pharmacological treatments—aducanumab, lecanemab, donanemab, gosuranemab, semorinemab, tilavonemab, zagotenemab, masupirdine, and sodium oligomannate—against placebo in patients with AD. Specifically, we focus on cognitive outcomes measured by the Clinical Dementia Rating–Sum of Boxes (CDR-SB), Alzheimer's Disease Assessment Scale–Cognitive Subscale (ADAS-cog), and the Mini-Mental State Examination (MMSE) with the aim of providing an integrated ranking of interventions and clarifying their relative therapeutic potential.^6–8^

## Methods

### Literature search strategy

A systematic literature search was conducted using the MEDLINE (via PubMed) database, covering studies from database inception to May 2025. We used a combination of Medical Subject Headings (MeSH) and free-text keywords, including “Alzheimer's disease”, “randomized controlled trial”, “CDR-SB”, “ADAS-cog”, “MMSE”, and “network meta-analysis”. Additional records were identified by manually screening the reference lists of relevant reviews and included articles. No restrictions were placed on language or publication status. The full, replicable search strategy for PubMed is provided in the Supplemental Material.

### Inclusion and exclusion criteria

#### Population

We included studies enrolling adults diagnosed with AD based on established clinical criteria (e.g., NIA-AA, DSM, or similar), with no restrictions on disease severity or duration.

#### Intervention

We included studies evaluating novel, mechanism-based pharmacological therapies for AD that have advanced to late-stage (Phase 2 or 3) randomized controlled trials (RCTs) as of May 2025. We focused on agents beyond traditional symptomatic treatments (e.g., cholinesterase inhibitors). This rationale led to the inclusion of nine specific agents representing the primary emerging therapeutic classes: Anti-amyloid (Aβ) monoclonal antibodies (aducanumab, lecanemab, donanemab); Anti-tau monoclonal antibodies (gosuranemab, semorinemab, tilavonemab, zagotenemab); Other novel mechanisms (masupirdine [a 5-HT6 antagonist] and sodium oligomannate [a gut-brain axis modulator]). Although mechanistically diverse, these interventions are considered “exchangeable” for this NMA because they (a) target the same broad patient population (mild-to-moderate AD), (b) are frequently tested against a common placebo comparator, and (c) are assessed using the same standardized cognitive outcomes (CDR-SB, ADAS-cog, MMSE), making them clinically relevant competitors in the current therapeutic development landscape.

#### Comparator

Comparisons with placebo or head-to-head evaluations between two or more of the eligible interventions were included.

#### Outcomes

To be eligible, studies were required to report at least one of the following cognitive measures: CDR-SB, ADAS-cog, or MMSE. Outcome data needed to be extractable in the form of mean, standard deviation (SD), and sample size for each treatment arm.

#### Study design

Only randomized controlled trials (RCTs) were eligible, including parallel, crossover, or factorial designs.

#### Exclusion criteria

Studies were excluded if they were non-randomized, investigated combination therapies without isolating the effects of individual agents, lacked key outcome data (including where data could not be obtained from authors), were duplicate publications, conference abstracts, or had critical methodological flaws such as undefined interventions or comparators.

### Outcome indicators

This network meta-analysis focused on three standardized cognitive outcomes commonly assessed in AD clinical trials. These were: (1) CDR-SB, evaluating cognitive and functional performance in daily life; (2) ADAS-cog, assessing memory, language, and praxis functions; (3) MMSE, reflecting global cognitive status.

Only change from baseline outcome scores were considered. When multiple time points were reported, data from the final follow-up or from the prespecified primary endpoint were extracted.

### Data extraction and quality assessment

Two reviewers independently screened titles and abstracts assessed full texts for eligibility and extracted data using a predefined extraction form. Extracted information included study characteristics (author, publication year, design), details of intervention and control groups, sample size, treatment duration, and change from baseline outcome scores (mean, standard deviation, and sample size). Discrepancies were resolved through discussion or third-party adjudication. When outcome data were missing or unclear, study authors were contacted for clarification.

The methodological quality of included studies was evaluated using the Cochrane Risk of Bias tool (RoB 2.0). Each study was assessed across seven domains: random sequence generation, allocation concealment, blinding of participants and personnel, blinding of outcome assessment, completeness of outcome data, selective reporting, and other potential biases. Each domain was rated as low, high, or unclear risk of bias.

### Statistical analysis

All statistical analyses were performed using Stata version 17.0 with the net meta package. A network plot was first constructed to visually represent the geometry of the evidence, illustrating how different interventions were compared across studies. Fixed- or random-effects models were applied depending on the degree of heterogeneity, quantified using the I^2^ statistic; values >50% were considered indicative of substantial heterogeneity, prompting the use of a random-effects model.

Consistency within the network was assessed by conducting node-splitting analyses, allowing for the comparison of direct and indirect evidence on specific treatment comparisons. If significant inconsistencies were detected (e.g., *p* < 0.1 from the node-splitting test), we planned to explore potential sources of heterogeneity by examining key trial-level characteristics, such as differences in baseline population severity or study duration. To rank the relative efficacy of the interventions, we calculated the Surface Under the Cumulative Ranking (SUCRA) values, with higher SUCRA scores indicating a greater likelihood of being the most effective treatment. Additionally, the probability of each treatment being ranked best (PrBest) was estimated from the ranking model. Sensitivity analyses were performed by excluding studies deemed at high risk of bias, thereby testing the stability of the primary findings. Finally, publication bias was evaluated through visual inspection of funnel plots, supplemented by Egger's regression test to statistically assess asymmetry.

## Results

### Identification of relevant studies

**Figure 1.**
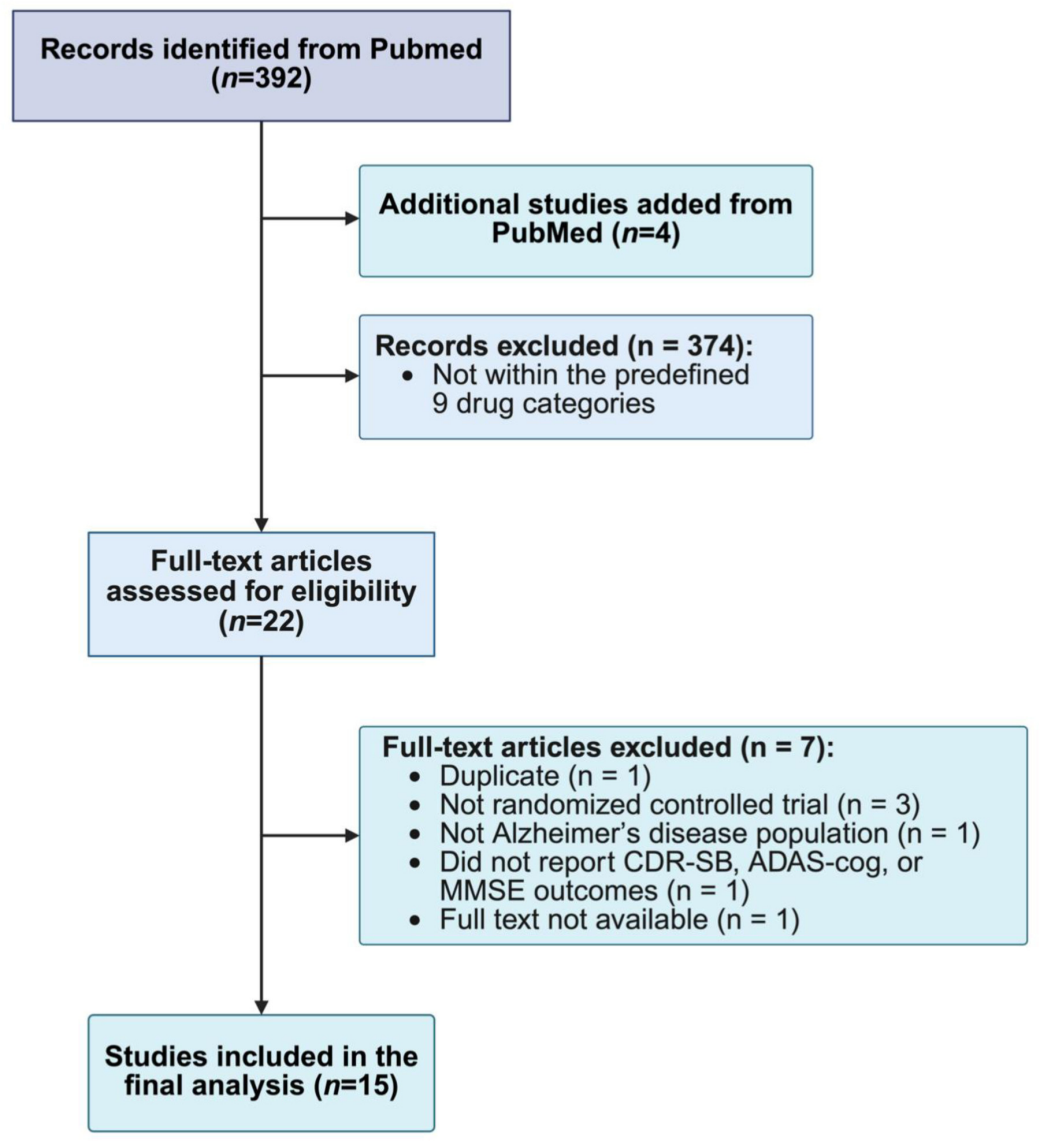
PRISMA flow diagram of the study selection process. A total of 392 records were identified from PubMed, with 4 additional studies added. After exclusions, 22 full-text articles were assessed for eligibility. Seven articles were excluded for reasons including duplicate records, not randomized controlled trials, non-AD population, lack of relevant cognitive outcomes (CDR-SB, ADAS-cog, MMSE), or unavailable full text. Finally, 15 studies were included in the analysis.

A total of 392 articles were initially collected according to the searching strategy on PubMed. After adding 4 additional studies from PubMed, 396 studies remained. Following title and abstract screening, 374 articles were excluded for clearly not meeting the inclusion criteria. A total of 22 were retained for full-text review. After detailed assessment, 7 articles were excluded for the following reasons: duplicate publication (n = 1), not randomized controlled trial (n = 3), not AD population (n = 1), absence of CDR-SB, ADAS-cog, or MMSE outcomes (n = 1), and full text not available (n = 1) (Figure 1).

Ultimately, 15 articles met the eligibility criteria and were included in the final network meta-analysis (Table 1). The literature screening process and results are presented in the Supplemental Material (Excel file).

### Risk of bias assessment and certainty of evidence

Risk of bias assessment showed that overall methodological quality was variable across the included studies. Specifically, two trials were judged to be at high risk of bias due to selective outcome reporting (domain D5). In addition, 7 studies were rated as having some concerns**,** while 5 studies were judged to be at low risk of bias across all assessed domains**.**

Regarding the certainty of evidence, treatment comparisons involving well-connected interventions such as aducanumab and lecanemab were supported by trials with low to moderate risk of bias. In contrast, comparisons involving zagotenemab and masupirdine were based on a small number of studies with methodological limitations. Overall, the quality of evidence was judged to be moderate for most primary outcomes, but lower for the MMSE-based secondary outcome.

Additional results, including SUCRA rankings, ranking probability distributions, and pairwise comparisons, are provided in the Supplemental Material (Supplemental Figures 1–6, Supplemental Tables 1–9).

### Network meta-analysis primary outcomes

#### CDR-SB score

**Figure 2.**
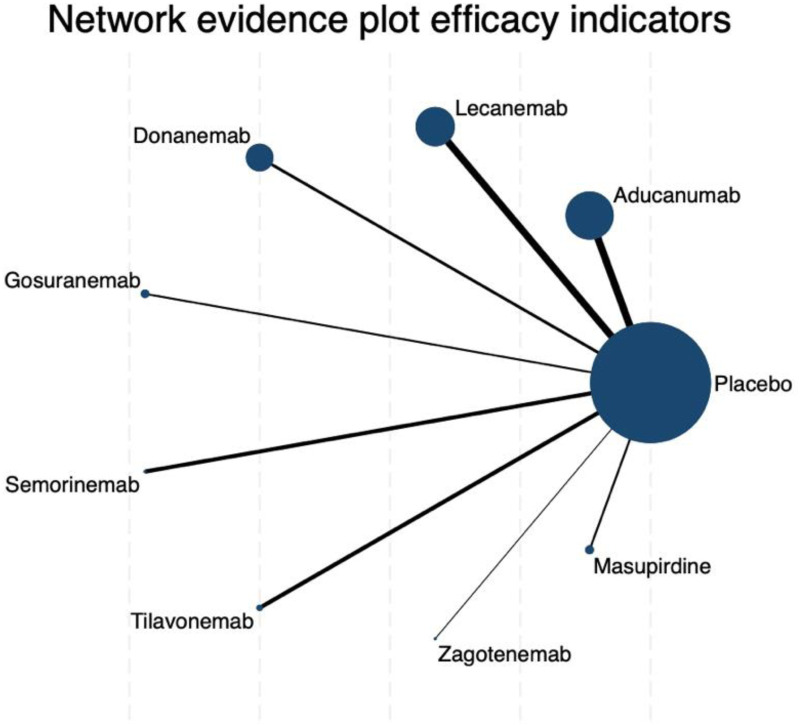
Network evidence plot of efficacy indicators for CDR-SB outcomes. Each node represents a treatment, with the node size proportional to the number of patients randomized to that treatment. The thickness of the connecting lines reflects the number of trials directly comparing the two connected treatments. Placebo is the most connected node, serving as the reference comparator for all other drugs.

The network meta-analysis of changes in CDR-SB revealed a well-connected evidence network, with placebo serving as the central node directly compared to all investigated interventions (Figure 2). The connections linking aducanumab and lecanemab to placebo were notably thicker, reflecting a greater number of direct comparisons and thus lending relatively more stability to their estimates. In contrast, the edges corresponding to zagotenemab and masupirdine were markedly thinner.

**Figure 3.**
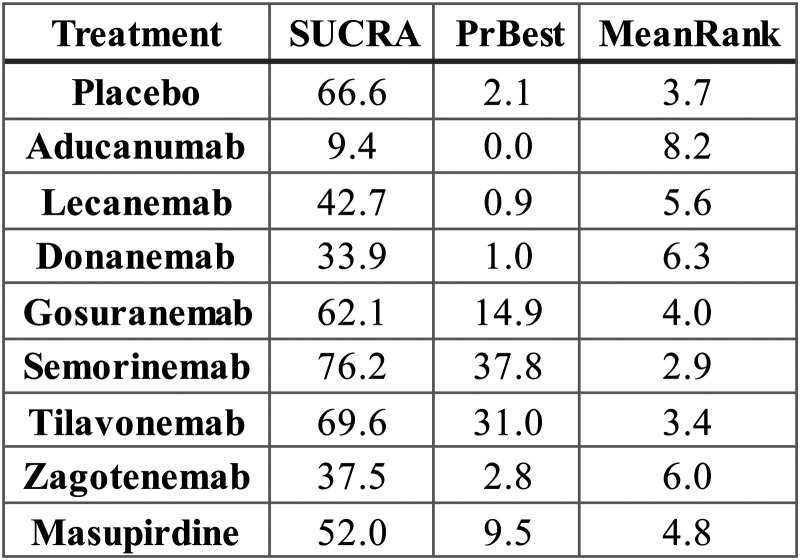
SUCRA values, probability of being best treatment (PrBest), and mean rank of each intervention for CDR-SB outcomes. Higher SUCRA values and lower mean rank indicate more favorable performance.

Based on SUCRA values, semorinemab ranked highest among all treatments for improving CDR-SB scores, with a SUCRA of 76.2, followed by tilavonemab (69.6), placebo (66.6), and gosuranemab (62.1) (Figure 3). It is noteworthy that placebo itself was positioned surprisingly high in the hierarchy, ranking third. Masupirdine (52.0), lecanemab (42.7), and donanemab (33.9) occupied intermediate positions, whereas zagotenemab (37.5) and aducanumab (9.4) were ranked lowest. Indeed, aducanumab demonstrated a virtually negligible probability of being the most effective treatment (PrBest = 0.0%) and was most frequently assigned to the eighth or ninth position across ranking iterations. In contrast, semorinemab and tilavonemab emerged as the most promising candidates, with probabilities of being ranked best of 37.8% and 31.0%, respectively. The cumulative rank probability distributions further underscored this pattern, showing that these two agents, along with placebo, were consistently concentrated in the top three ranks, while aducanumab's distribution clustered at the bottom (Supplemental Figure 1, Supplemental Table 1).

Despite the favorable ranking probabilities observed for semorinemab and tilavonemab, traditional pairwise meta-analyses painted a more cautious picture (Supplemental Table 2). The forest plots comparing mean differences in CDR-SB scores versus placebo indicated that, for nearly all treatments, the 95% confidence intervals crossed zero. Consequently, while the network rankings point toward a potential relative advantage for semorinemab and tilavonemab, the overall uncertainty remains considerable when viewed through the lens of traditional meta-analytic contrasts.

Sensitivity analyses and assessments of the model's underlying assumptions lent additional context to these findings. The funnel plot of effect sizes displayed a slight leftward asymmetry but remained broadly symmetrical, with Egger's regression tests yielding p-values of 0.054 (A versus C) and 0.066 (A versus B) (Supplemental Table 3), neither of which met conventional thresholds for statistical significance. These results do not provide strong evidence for the presence of publication bias, though the mild asymmetry warrants cautious interpretation (Supplemental Figure 2). Furthermore, node-splitting analyses for consistency revealed non-significant p-values, supporting the assumption of coherence between direct and indirect evidence within the network.

Taken together, these results show that among the pharmacologic agents evaluated, semorinemab and tilavonemab may hold relative promise for attenuating cognitive decline as measured by CDR-SB. Nevertheless, the lack of statistically significant differences in direct pairwise comparisons and the elevated placement of placebo within the ranking schema highlight the persistent uncertainty surrounding these interventions. Further large-scale, rigorously conducted randomized trials will be essential to more definitively ascertain their comparative efficacy.

#### ADAS-Cog score

**Figure 4.**
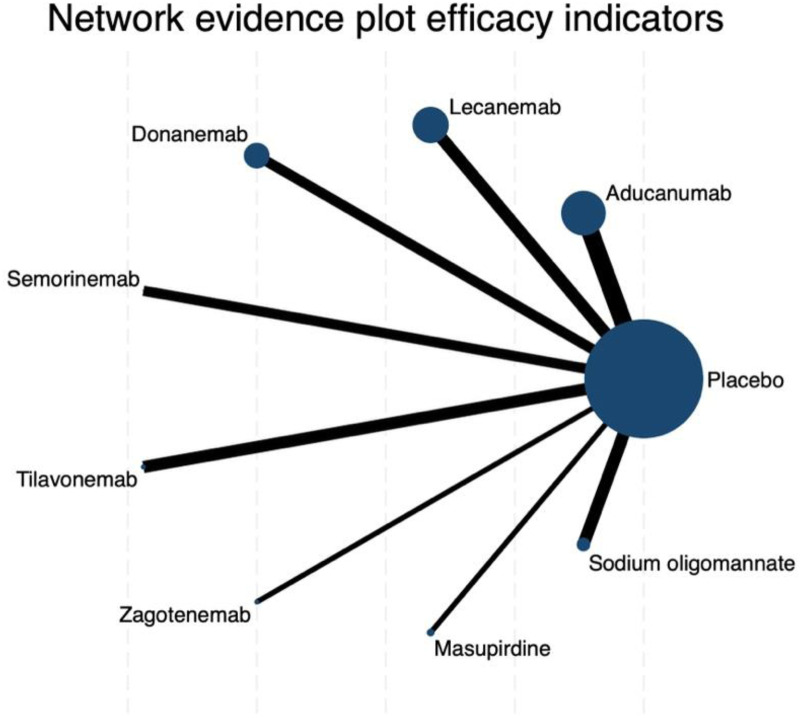
Network evidence plot of efficacy indicators for ADAS-cog outcomes. Node size represents the sample size per treatment and line thickness indicates the number of direct comparisons.

The network meta-analysis for changes in ADAS-cog scores revealed a star-shaped structure highly similar to that observed in the CDR-SB analysis. Placebo functioned as the central comparator node, directly connected to all nine active interventions, with particularly thick links to aducanumab, lecanemab, semorinemab, sodium oligomannate, and tilavonemab. This reflects the relatively larger evidence base for these treatments in placebo-controlled trials (Figure 4).

**Figure 5.**
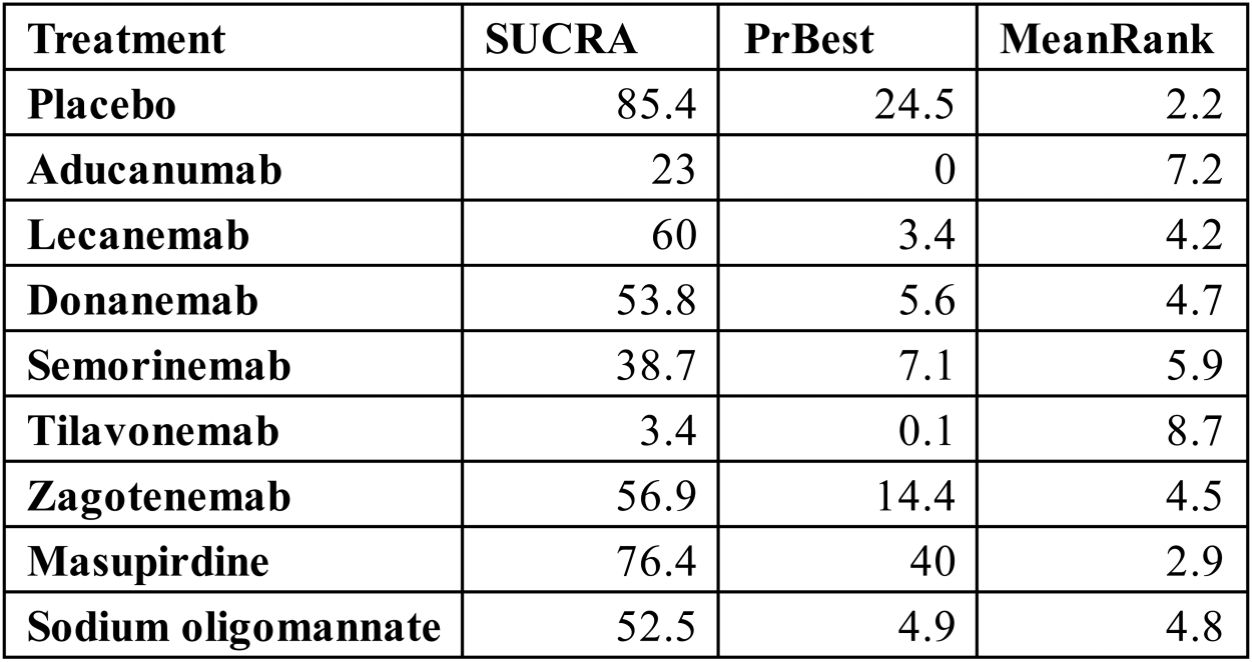
Surface under the cumulative ranking curve (SUCRA), probability of being the best treatment (PrBest), and mean rank of each intervention for ADAS-cog outcomes.

In terms of treatment ranking, placebo unexpectedly achieved the highest SUCRA value (85.4%), with a probability of being the best treatment (PrBest) of 24.5% and a mean rank of 2.2. Among active agents, masupirdine emerged as the most favorable intervention (SUCRA: 76.4%; PrBest: 40.0%; mean rank: 2.9), followed by lecanemab (SUCRA: 60.0%; PrBest: 3.4%) and zagotenemab (SUCRA: 56.9%; PrBest: 14.4%). By contrast, tilavonemab consistently ranked lowest (SUCRA: 3.4%), with an 82.1% probability of being the worst treatment, while aducanumab also demonstrated poor performance (SUCRA: 25.0%; PrBest: 0%; mean rank: 7.0). These findings highlight a striking divergence in relative efficacy probabilities, favoring placebo and masupirdine while reinforcing the unfavorable standing of tilavonemab and aducanumab (Figure 5).

The rank probability plots further substantiated these patterns. Placebo showed the highest overall likelihood of occupying top positions (rank 1–3), whereas tilavonemab had a dominant probability of being ranked last (Supplemental Figure 3). Masupirdine displayed the greatest probability of achieving rank 1 (40.0%), albeit with wide distribution across intermediate ranks (Supplemental Table 4).

Pairwise comparisons largely confirmed these observations. Most active interventions versus placebo yielded wide confidence intervals crossing zero, indicating a lack of statistically significant superiority. Notably, however, placebo demonstrated statistically significant advantages over certain active drugs. For instance, placebo was superior to tilavonemab (mean difference: 7.40; 95% CI: 2.66 to 12.14) and aducanumab (mean difference: 3.51; 95% CI: 1.63 to 5.39). These results account for the unusually high ranking of placebo in SUCRA analysis and underscore the limited efficacy of several monoclonal antibodies within this outcome domain (Supplemental Table 5).

Assessment of small-study effects revealed potential concerns. Funnel plots exhibited mild asymmetry, and Egger's tests detected significant publication bias for one comparison (A versus B, *p* = 0.0081), though other contrasts were not statistically significant (Supplemental Figure 4).

Furthermore, inconsistency analyses identified discrepancies between direct and indirect evidence, suggesting that the network's transitivity assumption may not be fully satisfied. These limitations indicate that the probabilistic rankings should be interpreted with caution (Supplemental Table 6).

In conclusion, the ADAS-cog analysis did not demonstrate robust superiority of any pharmacological intervention over placebo. Instead, placebo and masupirdine achieved the most favorable rankings, whereas tilavonemab and aducanumab consistently performed worst. These findings contrast with earlier results for other scales, highlighting both the heterogeneity of cognitive outcome measures and the substantial challenges in achieving reliable efficacy signals across AD clinical trials.

### Secondary outcome

**Figure 6.**
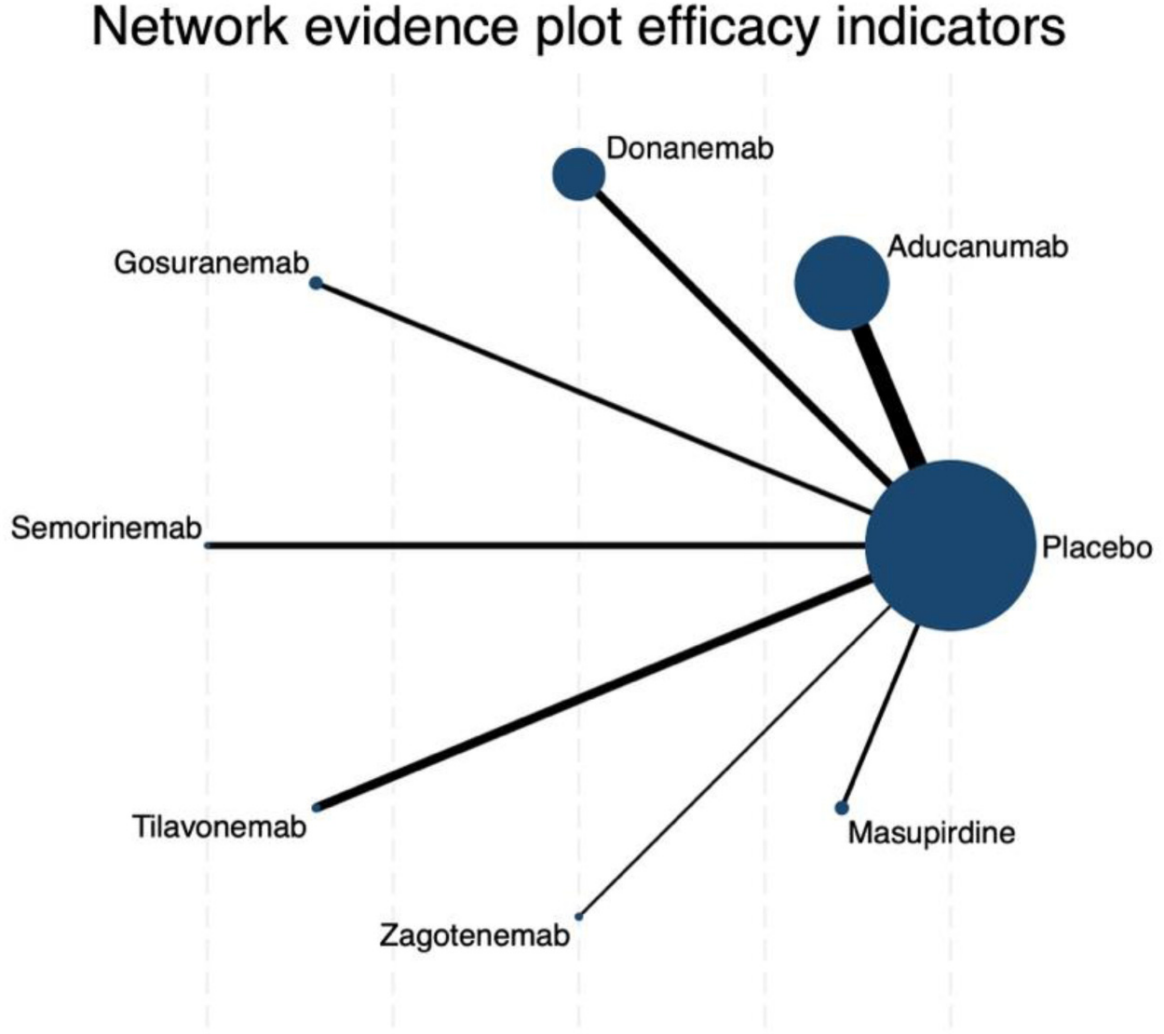
Network evidence plot of efficacy indicators for MMSE outcomes. Node size reflects the number of patients randomized to each treatment, while edge thickness represents the number of trials directly comparing connected interventions.

For the secondary outcome assessing changes in MMSE scores, the network geometry retained a star-shaped structure, with placebo serving as the central comparator directly connected to all active interventions. The thickest connections were observed between placebo and aducanumab as well as tilavonemab (Figure 6).

**Figure 7.**
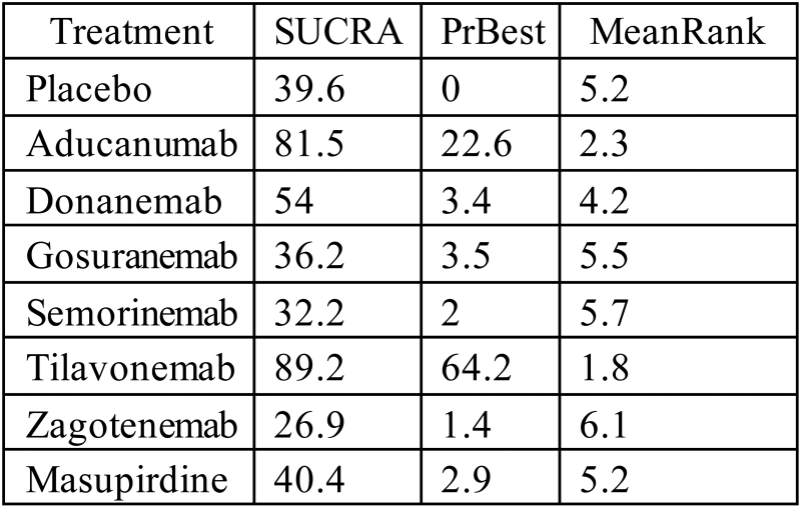
Surface under the cumulative ranking curve (SUCRA), probability of being the best treatment (PrBest), and mean rank of each intervention for MMSE outcomes.

Based on SUCRA values and ranking probabilities, the treatment hierarchy differed significantly from the primary outcomes. The most highly rated intervention was tilavonemab (SUCRA: 89.2%; PrBest: 64.2%; mean rank: 1.8), which was closely followed by aducanumab (SUCRA: 81.5%; PrBest: 22.6%; mean rank: 2.3). Donanemab's performance was intermediate (mean rank: 4.2; SUCRA: 54.0%) (Figure 7). On the other hand, all active medications, such as zagotenemab, gosuranemab, semorinemab, and masupirdine, as well as a placebo, were consistently found in the lower ranks. The rank-probability plots, which displayed the probability distribution for aducanumab peaking at rank two and for tilavonemab peaking at rank one, further corroborated these trends (Supplemental Figure 5, Supplemental Table 7).

The pairwise comparisons (Supplemental Table 8) revealed a more complex and subtle perspective. The only active treatment that demonstrated a statistically significant advantage over a placebo was aducanumab (mean difference −1.99, 95% CI – 3.68 to −0.3). The fact that tilavonemab failed to produce a statistically significant outcome even though it was the drug with the highest SUCRA ranking was particularly significant. Actually, compared to a placebo, its negative mean difference (mean difference −3.20, 95% CI −6.88 to 0.48) indicated a trend of worse performance. The confidence intervals for all other active interventions crossed zero, indicating no meaningful differences from placebo.

The model diagnostics, on the other hand, showed serious problems that make these efficacy findings very questionable. The funnel plot for the MMSE analysis exhibited significant asymmetry (Supplemental Figure 6), indicating that smaller studies generally reported larger effect sizes. Egger's test confirmed this finding by showing a statistically significant intercept (*p* = 0.016), which strongly suggested either publication bias or small-study effects (Supplemental Table 9). This discovery significantly affects the trustworthiness of the entire MMSE analysis and raises considerable doubt regarding the purported efficacy of aducanumab.

In summary, although an initial examination of the MMSE analysis indicated that tilavonemab and aducanumab were the most promising agents, with aducanumab displaying a relatively significant advantage, these conclusions lack credibility. The robust statistical evidence of publication bias strongly indicates that the observed positive effects, particularly for aducanumab, are likely a statistical artefact. Consequently, these findings must be regarded with extreme caution.

### Publication bias

Publication bias assessment revealed heterogeneous findings across different cognitive outcomes. For the primary outcome (CDR-SB) and the secondary outcome (ADAS-cog), funnel plots appeared largely symmetrical, and Egger's test did not identify statistically significant small-study effects (*p* > 0.05). These results suggest that the risk of publication bias for these measures is minimal, lending greater confidence to the pooled estimates.

In contrast, the MMSE outcome exhibited clear evidence of bias. The funnel plot showed marked asymmetry, with smaller studies disproportionately reporting larger treatment effects. This was corroborated by Egger's test (*p* = 0.0159), indicating significant small-study effects or selective reporting. Importantly, aducanumab—the only drug showing nominal statistical superiority over placebo on MMSE—may have had its apparent benefit exaggerated due to this bias. Moreover, MMSE is inherently less sensitive to subtle cognitive changes in early-stage AD and is prone to ceiling effects, further limiting the robustness of these findings.

Taken together, while the CDR-SB and ADAS-cog results appear methodologically reliable, the MMSE findings should be interpreted with caution. These observations underscore the need for more rigorous trial reporting, prospective registration, and larger-scale studies to mitigate selective publication and improve the reliability of cognitive outcome assessments in AD research.

## Discussion

In this network meta-analysis comparing nine pharmacological treatments for AD with placebo, no active drug demonstrated a robust or statistically significant cognitive benefit over placebo on the primary outcomes (CDR-SB and ADAS-cog). While two experimental therapies, semorinemab and tilavonemab (both targeting tau protein pathology), ranked highest in terms of probability of being the best treatment, their advantages were modest and not statistically significant in direct comparisons. The placebo showed unexpectedly strong performance, ranking near the top across multiple outcomes and even achieving the highest SUCRA value on ADAS-cog, exceeding all active treatments. This finding underscores the modest and uncertain efficacy signals of current agents. In contrast, aducanumab produced inconsistent results. It ranked lowest on CDR-SB and ADAS-cog but appeared near the top on MMSE, where it was the only drug demonstrating nominal statistical superiority over placebo. However, this apparent advantage is undermined by evidence of publication bias (Egger's test *p* = 0.0159), casting doubt on the reliability of MMSE-derived findings. Taken together, these results highlight the limited and inconsistent cognitive benefits of available therapies, the pronounced role of placebo responses in AD trials, and the methodological vulnerabilities of different cognitive assessment tools.

Several explanations may account for these findings. First, most active drugs do not outperform placebo in statistically significant ways, largely due to small or non-significant effect sizes. Their confidence intervals frequently crossed zero, thereby weakening their ranking. Second, certain interventions were evaluated in only a limited number of trials, often with large standard errors and heterogeneous results, further reducing confidence in their estimated benefits. Third, placebo performance remained consistent despite the absence of active pharmacological effects. In a probabilistic ranking model, such consistency of performance may result in a higher rank when other treatments perform poorly or inconsistently.

An additional methodological limitation across several included trials relates to the use and reporting of placebo comparators. Future AD trials may benefit from comparing investigational agents against optimized standard-of-care therapy rather than placebo alone, in line with best practices in clinical trial design, to improve interpretability and clinical relevance of efficacy estimates.

Meanwhile, the relative success of tau-targeting antibodies (semorinemab and tilavonemab) is highly supported by the accumulating evidence that AD tau pathology correlates more closely with cognitive decline than amyloid burden.^19^ Nonetheless, the small, non-significant effect sizes observed here suggest that current tau antibodies may not yet achieve sufficient target engagement or brain penetration to translate into robust clinical benefits.^20^

More critically, our analysis detected a statistically significant Egger's test result for the MMSE outcome (*p* = 0.0159), providing strong evidence of publication bias or small-study effects. This elevates the concern from a methodological limitation to a direct challenge to the validity of the observed aducanumab result. The observed “benefit” is likely a statistical artifact, not a true clinical effect, amplified by selective reporting or an overrepresentation of smaller, positive studies. This conclusion aligns with the broader scientific and regulatory controversy surrounding the drug. The European Medicines Agency (EMA) initially refused marketing authorization for aducanumab, citing conflicting trial results and an unproven link between its biomarker effect (amyloid reduction) and meaningful clinical improvement.^2^ More recently, the EMA has approved other anti-amyloid monoclonal antibodies, including lecanemab and donanemab, following additional evidence of modest clinical benefit. Importantly, however, this regulatory change does not resolve the specific concerns surrounding aducanumab. In our analysis, the apparent MMSE benefit of aducanumab coincides with clear evidence of publication bias and the known limitations of MMSE as a cognitive outcome measure. Taken together, the convergence of a flawed instrument, evidence of statistical bias from our analysis, and a negative regulatory review builds an overwhelming case to interpret aducanumab's apparent MMSE benefit with extreme uncertainty.

Methodologically, this study highlights the value of NMA for comparing multiple interventions when head-to-head trials are scarce. By integrating direct and indirect evidence, NMA enables comprehensive treatment ranking, though several limitations remain. NMA findings are typically presented using statistical metrics like SUCRA, indicating the likelihood of each treatment being the most favorable option.^21^ It is important to note that an SUCRA number nearing 100% indicates a higher likelihood of the therapy ranking highly in efficacy, but it does not necessarily correlate with more significant clinical benefits or more robust evidence.^22^ A high ranking does not indicate a definitive superiority in effectiveness nor suggest an especially high level of confidence.^23^ Therefore, when treatment effects are just slightly distinct or the quality of evidence is insufficient, relying only on SUCRA rankings for decision-making may be misleading.^24^ Such assessments should be augmented by a thorough examination that includes real effect magnitude and variability.

First, the evidence network was uneven. Certain treatment nodes, such as zagotenemab and masupirdine, were supported by only a small number of trials, reducing the precision and reliability of their estimated effects. Second, the MMSE outcome appeared particularly vulnerable to bias. Funnel plot asymmetry and a statistically significant Egger's test (*p* = 0.0159) indicated the presence of publication bias or small-study effects. This strongly undermines confidence in the apparent benefit of aducanumab, which was largely driven by MMSE findings. Third, consistency tests indicated significant inconsistency between the aducanumab and semorinemab comparison groups, cautioning against overinterpretation of MMSE results. Additionally, heterogeneity in cognitive outcomes and inconsistencies in trial duration across studies may introduce unmeasured variability.

Beyond the statistical limitations of the NMA, the interpretation of efficacy in the included trials requires caution due to fundamental design shortcomings highlighted in recent critical reviews. The treatment of adverse events may induce enrichment bias. Patients with low antibody tolerance, generally due to amyloid-related imaging abnormalities (ARIA), may be excluded from efficacy assessments or discontinue treatment early. This favors a ‘super-responder’ population that tolerates the treatment, perhaps increasing efficacy relative to the general AD population.^25^ Additionally, most trials did not strictly control for changes in conventional background therapy, making it difficult to isolate the true pharmacological effect of the investigational agents from variations in standard care.^25^

Taken together, these limitations highlight the need for cautious interpretation of treatment rankings and underscore the importance of larger designed trials with standardized cognitive endpoints.

Consistent with previous meta-analyses, our results show that the active interventions investigated did not show statistically significant benefits over placebo on the primary outcomes (CDR-SB and ADAS-cog), highlighting the limited effectiveness of existing pharmacotherapies for AD. The relatively high rankings of the tau-targeting antibodies semorinemab and tilavonemab in our analysis align with reports suggesting that showing both agents offer comparable or superior cognitive improvement to placebo with favorable safety profiles. The incidence of adverse events (AEs), serious adverse events (SAEs), falls, and urinary tract infections was lower in the tilavonemab group than in the placebo group, and the differences were statistically significant.^4^ The favorable ranking of semorinemab in our network meta-analysis may reflect its target engagement and downstream pharmacodynamic effects, as supported by recent biomarker studies. In particular, Schauer et al.^26^ demonstrated that semorinemab reduced cerebrospinal fluid levels of tau-related pathology markers, including phosphorylated tau and neurogranin, which may underlie its potential to slow cognitive decline in AD. These findings suggest that the high SUCRA rankings of semorinemab and tilavonemab in our analysis may reflect modest but genuine clinical effects of tau-targeted approaches.

Interestingly, our secondary analysis of MMSE revealed a markedly improved ranking for aducanumab, in contrast to its poor performance on CDR-SB and ADAS-cog. Mechanistically, aducanumab has been shown to distinguish monomers from oligomers or fibrillar aggregates based on its weak monovalent affinity, rapid binding kinetics, and strong affinity for epitope-rich aggregates.^27,28^ Despite this pharmacological rationale, regulatory evaluations have raised substantial concerns. The U.S. Food and Drug Administration (FDA) has reported significantly higher incidences of ARIA-edema and ARIA-hemorrhage, collectively referred to as ARIA, in patients treated with aducanumab.^25^ Beyond these acute events, emerging evidence suggests a paradoxical ‘therapeutic’ consequence: accelerated brain volume loss.^29^ Long-term assessment of patients treated with anti-amyloid immunotherapies has revealed significant whole-brain atrophy and ventricular enlargement compared to placebo.^30^ These structural changes raise critical questions about whether the clearance of amyloid plaques comes at the cost of neuronal integrity.^31,32^ At the time of earlier regulatory assessments, the European Medicines Agency (EMA) has stated that aducanumab has not yet demonstrated clear clinical efficacy or an acceptable safety profiles patients with early-stage AD.^33^ Since then, the EMA has approved other anti-amyloid monoclonal antibodies, including lecanemab and donanemab, following the availability of additional trail data indicating modest clinical benefit. Biogen's ENVISION study, a validation study assessing the safety and efficacy of long-term treatment after discontinuation, is expected to be completed by 2026.^33^ In this study, due to the relatively small number of studies included in our MMSE analysis, funnel plot asymmetry and a significant Egger's test suggest the possibility of small-study or publication bias. Egger regression is commonly used to assess funnel plot asymmetry, but its accuracy decreases when fewer than 10 trials are available. Thus, the favorable ranking of aducanumab on MMSE should be interpreted with caution, and more robust, adequately powered randomized trials are needed to clarify its true effect.

Although this may seem counterintuitive, high placebo scores in our analysis are not unprecedented in AD trials. Recent meta-analyses of trials targeting AD and mild cognitive impairment have confirmed strong placebo responses, which are typically attributed to expectation effects,^34^ researcher-participant interaction, regression to the mean effects, and repeated cognitive testing.^35^ These findings suggest that the unexpected placebo effects observed in this study likely reflect inherent factors inherent to the trial itself rather than genuine therapeutic benefits.

This study, along with most of the included trials, used PET imaging markers (such as changes in cerebral amyloid or tau burden) as a surrogate endpoint. However, a decrease in the imaging signal of pathological burden does not indicate a complete absence of the disease pathology, and a definitive causal relationship between imaging enhancement and clinical benefit for patients remains unestablished. Prior research has demonstrated that a simple decrease in cerebral amyloid beta burden is insufficient to significantly prevent cognitive decline over follow-up periods.^36^ Furthermore, the reliance on structural MRI and amyloid-PET as primary biomarkers fails to capture the functional metabolic state of the brain following treatment.^37^ There is a notable scarcity of data utilizing fluorodeoxyglucose-positron emission tomography (FDG-PET) to assess regional metabolic activity in areas affected by ARIA or atrophy.^29^ Currently, no interventions targeting amyloid beta have shown significant improvements in clinical efficacy.^38,39^ This indicates that efficacy evidence derived mainly from PET surrogate endpoints requires careful analysis.

Despite encouraging changes in biomarkers, several tau-targeting monoclonal antibodies, including semorinemab, have failed to demonstrate significant clinical benefits in late-stage clinical trials. The limited efficacy of tau-targeting antibodies, such as semorinemab and tilavonemab, likely stems from fundamental mechanistic barriers.^40,41^ Pathological tau tangles are primarily located intracellularly, posing a significant challenge for conventional monoclonal antibodies. Due to their substantial molecular size, these antibodies cannot readily penetrate the neuronal cell membrane to access and clear intraneuronal aggregates.^42^ Current approaches largely rely on the hypothesis of intercepting extracellular ‘seed’ tau (the “interceptor” Hypothesis) to prevent cell-to-cell propagation.^43^ However, as our results indicate, targeting this extracellular fraction alone may be insufficient to halt clinical decline once intracellular pathology is established. This highlights a critical limitation in the current design of passive immunotherapies: without effective intracellular delivery mechanisms, antibodies may fail to engage the most relevant pathological targets. Additionally, recent quantitative systems pharmacology modelling suggests that semorinemab has relatively low target binding rates in cerebrospinal fluid, which may be related to its lower affinity and shorter half-life in extended dosing regimens.^44^ To be more specific, antibodies targeting the N-terminal region of tau protein may successfully bind to soluble tau protein in cerebrospinal fluid but fail to affect pathological tau protein or intracellular aggregates in the brain.^44^ Future studies should consider alternative tau antigen epitopes, improve antibody pharmacokinetic properties, or adopt delivery strategies that enhance brain penetration.

Meanwhile, current animal and *in vitro* models may not adequately predict the functional efficacy of antibodies targeting tau protein. Induced pluripotent stem cell (iPSC)-derived neurons and brain organoids offer promising human-relevant platforms for preclinical evaluation.^45^ These models can be used to test antigen-specific binding, cellular uptake, and downstream tau protein clearance efficiency under conditions similar to those in humans.^46^ Future research should combine human neuronal models derived from iPSCs with pharmacokinetic analysis to design more effective tau immunotherapies that can penetrate the blood-brain barrier.^47^

### Conclusion

This network meta-analysis of nine AD drugs found that none provided a clinically meaningful cognitive benefit over a placebo.

Tau-targeting antibodies showed only marginal, statistically insignificant potential. The apparent benefit of another drug, aducanumab, was found to be highly unreliable due to strong evidence of publication bias and limitations of the cognitive test used.

Overall, these findings underscore the limitations of the current AD therapeutic landscape and highlight the significant influence of the placebo response in clinical trials. Future research should include larger, high-quality randomized controlled trials to validate these comparative efficacy trends and detect any meaningful treatment effects. Additionally, greater use of advanced human neuronal models, such as induced pluripotent stem cell (iPSC)-derived neurons and brain organoids, is recommended to better elucidate the mechanistic efficacy of tau-targeting therapies. Such approaches will help inform the development of more effective treatments for AD and ensure that promising therapeutic strategies are rigorously evaluated before clinical adoption.

## Supplemental Material

sj-docx-2-alr-10.1177_25424823261422205 - Supplemental material for Network meta-analysis of the efficacy of nine drugs for cognitive function in patients with Alzheimer's disease Supplemental material, sj-docx-2-alr-10.1177_25424823261422205 for Network meta-analysis of the efficacy of nine drugs for cognitive function in patients with Alzheimer's disease by Shanshan Huang and Yunyun Guo in Journal of Alzheimer's Disease Reports

sj-xlsx-3-alr-10.1177_25424823261422205 - Supplemental material for Network meta-analysis of the efficacy of nine drugs for cognitive function in patients with Alzheimer's disease Supplemental material, sj-xlsx-3-alr-10.1177_25424823261422205 for Network meta-analysis of the efficacy of nine drugs for cognitive function in patients with Alzheimer's disease by Shanshan Huang and Yunyun Guo in Journal of Alzheimer's Disease Reports

sj-xlsx-4-alr-10.1177_25424823261422205 - Supplemental material for Network meta-analysis of the efficacy of nine drugs for cognitive function in patients with Alzheimer's disease Supplemental material, sj-xlsx-4-alr-10.1177_25424823261422205 for Network meta-analysis of the efficacy of nine drugs for cognitive function in patients with Alzheimer's disease by Shanshan Huang and Yunyun Guo in Journal of Alzheimer's Disease Reports

## References

[1] Mintun MA Lo AC Duggan Evans C , et al. Donanemab in early Alzheimer’s disease. N Engl J Med 2021; 384: 1691–1704. 33720637 10.1056/NEJMoa2100708

[2] Budd Haeberlein S Aisen PS Barkhof F , et al. Two randomized phase 3 studies of aducanumab in early Alzheimer’s disease. J Prev Alzheimers Dis 2022; 9: 197–210. 35542991 10.14283/jpad.2022.30

[3] van Dyck CH Swanson CJ Aisen P , et al. Lecanemab in early Alzheimer’s disease. N Engl J Med 2023; 388: 9–21. 36449413 10.1056/NEJMoa2212948

[4] Cai W Zhang H Wu Y , et al. Comparative the efficacy and safety of gosuranemab, semorinemab, tilavonemab, and zagotenemab in patients with Alzheimer’s disease: a systematic review and network meta-analysis of randomized controlled trials. Front Aging Neurosci 2025; 16: 1465871. 39945003 10.3389/fnagi.2024.1465871 PMC11814219

[5] Monteiro C Toth B Brunstein F , et al. Randomized phase II study of the safety and efficacy of semorinemab in participants with mild-to-moderate Alzheimer disease: lauriet. Neurology 2023; 101: e1391–e1401. 10.1212/WNL.0000000000207663 PMC10573141 37643887

[6] Straten G Saur R Laske C , et al. Influence of lithium treatment on GDNF serum and CSF concentrations in patients with early Alzheimer’s disease. Curr Alzheimer Res 2011; 8: 853–859. 21875410 10.2174/156720511798192754

[7] Ballard C Banister C Khan Z , et al. Evaluation of the safety, tolerability, and efficacy of pimavanserin versus placebo in patients with Alzheimer’s disease psychosis: a phase 2, randomised, placebo-controlled, double-blind study. Lancet Neurol 2018; 17: 213–222. 29452684 10.1016/S1474-4422(18)30039-5

[8] Chen C Katayama S Lee J-H , et al. Clarity AD: Asian regional analysis of a phase III trial of lecanemab in early Alzheimer’s disease. J Prev Alzheimers Dis 2025; 12: 100160. 40189473 10.1016/j.tjpad.2025.100160 PMC12184022

[9] Dhadda S Kanekiyo M Li D , et al. Consistency of efficacy results across various clinical measures and statistical methods in the lecanemab phase 2 trial of early Alzheimer’s disease. Alzheimers Res Ther 2022; 14: 182. 36482412 10.1186/s13195-022-01129-x PMC9733166

[10] Florian H Wang D Arnold SE , et al. Tilavonemab in early Alzheimer’s disease: results from a phase 2, randomized, double-blind study. Brain 2023; 146: 2275–2284. 36730056 10.1093/brain/awad024 PMC10232284

[11] Shulman M Kong J O’Gorman J , et al. TANGO: a placebo-controlled randomized phase 2 study of efficacy and safety of the anti-tau monoclonal antibody gosuranemab in early Alzheimer’s disease. Nat Aging 2023; 3: 1591–1601. 38012285 10.1038/s43587-023-00523-w PMC10724064

[12] Sims JR Zimmer JA Evans CD , et al. Donanemab in early symptomatic Alzheimer disease: the TRAILBLAZER-ALZ 2 randomized clinical trial. JAMA 2023; 330: 512–527. 37459141 10.1001/jama.2023.13239 PMC10352931

[13] Swanson CJ Zhang Y Dhadda S , et al. A randomized, double-blind, phase 2b proof-of-concept clinical trial in early Alzheimer’s disease with lecanemab, an anti-Aβ protofibril antibody. Alzheimers Res Ther 2021; 13: 80. 33865446 10.1186/s13195-021-00813-8 PMC8053280

[14] Toda Y Iwatsubo T Nakamura Y , et al. Japanese Subgroup analyses from EMERGE and ENGAGE, phase 3 clinical trials of aducanumab in patients with early Alzheimer’s disease. J Prev Alzheimers Dis 2024; 11: 1260–1269. 39350371 10.14283/jpad.2024.106 PMC11436400

[15] Nirogi R Ieni J Goyal VK , et al. Effect of masupirdine (SUVN-502) on cognition in patients with moderate Alzheimer’s disease: a randomized, double-blind, phase 2, proof-of-concept study. Alzheimers Dement (N Y) 2022; 8: e12307. 10.1002/trc2.12307 PMC9157584 35662833

[16] Fleisher AS Munsie LM Perahia DGS , et al. Assessment of efficacy and safety of zagotenemab: results from PERISCOPE-ALZ, a phase 2 study in early symptomatic Alzheimer disease. Neurology 2024; 102: e208061. 10.1212/WNL.0000000000208061 PMC11067698 38386949

[17] Wang T Chen W Xu W , et al. A phase II randomized trial of sodium oligomannate in Alzheimer’s dementia. Alzheimers Res Ther 2020; 12: 110. 32928279 10.1186/s13195-020-00678-3 PMC7489025

[18] Xiao S Chan P Wang T , et al. A 36-week multicenter, randomized, double-blind, placebo-controlled, parallel-group, phase 3 clinical trial of sodium oligomannate for mild-to-moderate Alzheimer’s dementia. Alzheimers Res Ther 2021; 13: 62. 33731209 10.1186/s13195-021-00795-7 PMC7967962

[19] La Joie R Visani AV Baker SL , et al. Prospective longitudinal atrophy in Alzheimer’s disease correlates with the intensity and topography of baseline tau-PET. Sci Transl Med 2020; 12: eaau5732. 10.1126/scitranslmed.aau5732 PMC7035952 31894103

[20] Cummings JL Gonzalez MI Pritchard MC , et al. The therapeutic landscape of tauopathies: challenges and prospects. Alzheimers Res Ther 2023; 15: 168. 37803386 10.1186/s13195-023-01321-7 PMC10557207

[21] Salanti G Ades AE Ioannidis JPA . Graphical methods and numerical summaries for presenting results from multiple-treatment meta-analysis: an overview and tutorial. J Clin Epidemiol 2011; 64: 163–171. 20688472 10.1016/j.jclinepi.2010.03.016

[22] Mbuagbaw L Rochwerg B Jaeschke R , et al. Approaches to interpreting and choosing the best treatments in network meta-analyses. Syst Rev 2017; 6: 79. 28403893 10.1186/s13643-017-0473-z PMC5389085

[23] Trinquart L Attiche N Bafeta A , et al. Uncertainty in treatment rankings: reanalysis of network meta-analyses of randomized trials. Ann Intern Med 2016; 164: 666–673. 27089537 10.7326/M15-2521

[24] Brignardello-Petersen R Mustafa RA Siemieniuk RAC , et al. GRADE Approach to rate the certainty from a network meta-analysis: addressing incoherence. J Clin Epidemiol 2019; 108: 77–85. 30529648 10.1016/j.jclinepi.2018.11.025

[25] Høilund-Carlsen PF Revheim M-E Alavi A , et al. FDG PET (and MRI) for monitoring immunotherapy in Alzheimer disease. Clin Nucl Med 2023; 48: 689–691. 37314733 10.1097/RLU.0000000000004710 PMC10317300

[26] Schauer SP Toth B Lee J , et al. Pharmacodynamic effects of semorinemab on plasma and CSF biomarkers of Alzheimer’s disease pathophysiology. Alzheimers Dement 2024; 20: 8855–8866. 39513754 10.1002/alz.14346 PMC11667501

[27] Ferrero J Williams L Stella H , et al. First-in-human, double-blind, placebo-controlled, single-dose escalation study of aducanumab (BIIB037) in mild-to-moderate Alzheimer’s disease. Alzheimers Dement (N Y) 2016; 2: 169–176. 29067304 10.1016/j.trci.2016.06.002 PMC5651340

[28] Arndt JW Qian F Smith BA , et al. Structural and kinetic basis for the selectivity of aducanumab for aggregated forms of amyloid-β. Sci Rep 2018; 8: 6412. 29686315 10.1038/s41598-018-24501-0 PMC5913127

[29] Alves F Kalinowski P Ayton S . Accelerated brain volume loss caused by anti-β-amyloid drugs: a systematic review and meta-analysis. Neurology 2023; 100: e2114–e2124. 10.1212/WNL.0000000000207156 PMC10186239 36973044

[30] Belder CRS Boche D Nicoll JAR , et al. Brain volume change following anti-amyloid β immunotherapy for Alzheimer’s disease: amyloid-removal-related pseudo-atrophy. Lancet Neurol 2024; 23: 1025–1034. 39304242 10.1016/S1474-4422(24)00335-1

[31] Hampel H Hardy J Blennow K , et al. The amyloid-β pathway in Alzheimer’s disease. Mol Psychiatry 2021; 26: 5481–5503. 34456336 10.1038/s41380-021-01249-0 PMC8758495

[32] Barkhof F Knopman DS . Brain shrinkage in anti-β-amyloid Alzheimer trials: neurodegeneration or pseudoatrophy? Neurology 2023; 100: 941–942. 36973045 10.1212/WNL.0000000000207268

[33] Vaz M Silva V Monteiro C , et al. Role of aducanumab in the treatment of Alzheimer’s disease: challenges and opportunities. Clin Interv Aging 2022; 17: 797–810. 35611326 10.2147/CIA.S325026 PMC9124475

[34] Colagiuri B Schenk LA Kessler MD , et al. The placebo effect: from concepts to genes. Neuroscience 2015; 307: 171–190. 26272535 10.1016/j.neuroscience.2015.08.017 PMC5367890

[35] Goldberg TE Harvey PD Wesnes KA , et al. Practice effects due to serial cognitive assessment: implications for preclinical Alzheimer’s disease randomized controlled trials. Alzheimers Dement (Amst) 2015; 1: 103–111. 27239497 10.1016/j.dadm.2014.11.003 PMC4876902

[36] Zhang Y Chen H Li R , et al. Amyloid β-based therapy for Alzheimer’s disease: challenges, successes and future. Signal Transduct Target Ther 2023; 8: 248. 37386015 10.1038/s41392-023-01484-7 PMC10310781

[37] Chételat G Arbizu J Barthel H , et al. Amyloid-PET and 18F-FDG-PET in the diagnostic investigation of Alzheimer’s disease and other dementias. Lancet Neurol 2020; 19: 951–962. 33098804 10.1016/S1474-4422(20)30314-8

[38] Walsh S Merrick R Richard E , et al. Lecanemab for Alzheimer’s disease. Br Med J 2022; 379: o3010. 10.1136/bmj.o3010 36535691

[39] Ebell MH Barry HC Baduni K , et al. Clinically important benefits and harms of monoclonal antibodies targeting amyloid for the treatment of Alzheimer disease: a systematic review and meta-analysis. Ann Fam Med 2024; 22: 50–62. 38253509 10.1370/afm.3050 PMC11233076

[40] Dam T Boxer AL Golbe LI , et al. Safety and efficacy of anti-tau monoclonal antibody gosuranemab in progressive supranuclear palsy: a phase 2, randomized, placebo-controlled trial. Nat Med 2021; 27: 1451–1457. 34385707 10.1038/s41591-021-01455-x

[41] Teng E Manser PT Pickthorn K , et al. Safety and efficacy of semorinemab in individuals with prodromal to mild Alzheimer disease: a randomized clinical trial. JAMA Neurol 2022; 79: 758–767. 35696185 10.1001/jamaneurol.2022.1375 PMC9194753

[42] Sigurdsson EM . Tau immunotherapies for Alzheimer’s disease and related tauopathies: progress and potential pitfalls. J Alzheimers Dis 2018; 64: S555–S565. 10.3233/JAD-179937 PMC6171771 29865056

[43] Gibbons GS Lee VMY Trojanowski JQ . Mechanisms of cell-to-cell transmission of pathological tau. JAMA Neurol 2019; 76: 101–108. 30193298 10.1001/jamaneurol.2018.2505 PMC6382549

[44] Geerts H Bergeler S Walker M , et al. Analysis of clinical failure of anti-tau and anti-synuclein antibodies in neurodegeneration using a quantitative systems pharmacology model. Sci Rep 2023; 13: 14342. 37658103 10.1038/s41598-023-41382-0 PMC10474108

[45] Shimada H Sato Y Sasaki T , et al. A next-generation iPSC-derived forebrain organoid model of tauopathy with tau fibrils by AAV-mediated gene transfer. Cell Rep Methods 2022; 2: 100289. 36160042 10.1016/j.crmeth.2022.100289 PMC9499998

[46] Bassil R Shields K Granger K , et al. Improved modeling of human AD with an automated culturing platform for iPSC neurons, astrocytes and microglia. Nat Commun 2021; 12: 5220. 34471104 10.1038/s41467-021-25344-6 PMC8410795

[47] Cheng C Reis SA Adams ET , et al. High-content image-based analysis and proteomic profiling identifies tau phosphorylation inhibitors in a human iPSC-derived glutamatergic neuronal model of tauopathy. Sci Rep 2021; 11: 17029. 34426604 10.1038/s41598-021-96227-5 PMC8382845

