# Supplementary material for: Network meta-analysis of the efficacy of nine drugs for cognitive function in patients with Alzheimer's disease
Source: J Alzheimers Dis Rep. 2026 Feb 6;10:25424823261422205. doi: 10.1177/25424823261422205 (PMC13039041; doi:10.1177/25424823261422205)
Supplement: sj-docx-2-alr-10.1177_25424823261422205 - Supplemental material for Network meta-analysis of the efficacy of nine drugs for cognitive function in patients with Alzheimer's disease [file sj-docx-2-alr-10.1177_25424823261422205.docx]

**Supplemental Material**

**Network meta-analysis of the efficacy of nine drugs for cognitive function in patients with Alzheimer’s disease**

**Full Search Strategy for PubMed**

"alzheimer disease"[MeSH Terms] OR "alzheimer s disease"[Title/Abstract]) AND ("Aducanumab"[Title/Abstract] OR "Lecanemab"[Title/Abstract] OR "Donanemab"[Title/Abstract] OR "Gosuranemab"[Title/Abstract] OR "Semorinemab"[Title/Abstract] OR "Tilavonemab"[Title/Abstract] OR "Zagotenemab"[Title/Abstract] OR "Masupirdine"[Title/Abstract] OR "sodium oligomannate"[Title/Abstract] OR "placebo"[Title/Abstract]) AND ("MMSE"[Title/Abstract] OR "CDR-SB"[Title/Abstract] OR "ADAS-cog"[Title/Abstract]) AND ((("random allocation"[MeSH Terms] OR ("random"[All Fields] AND "allocation"[All Fields]) OR "random allocation"[All Fields] OR "randomization"[All Fields] OR "randomized"[All Fields] OR "random"[All Fields] OR "randomisation"[All Fields] OR "randomisations"[All Fields] OR "randomise"[All Fields] OR "randomised"[All Fields] OR "randomising"[All Fields] OR "randomizations"[All Fields] OR "randomize"[All Fields] OR "randomizes"[All Fields] OR "randomizing"[All Fields] OR "randomness"[All Fields] OR "randoms"[All Fields]) AND "controlled clinical trial"[Publication Type]) OR "RCT"[Title/Abstract])

**CDR-SB**

**
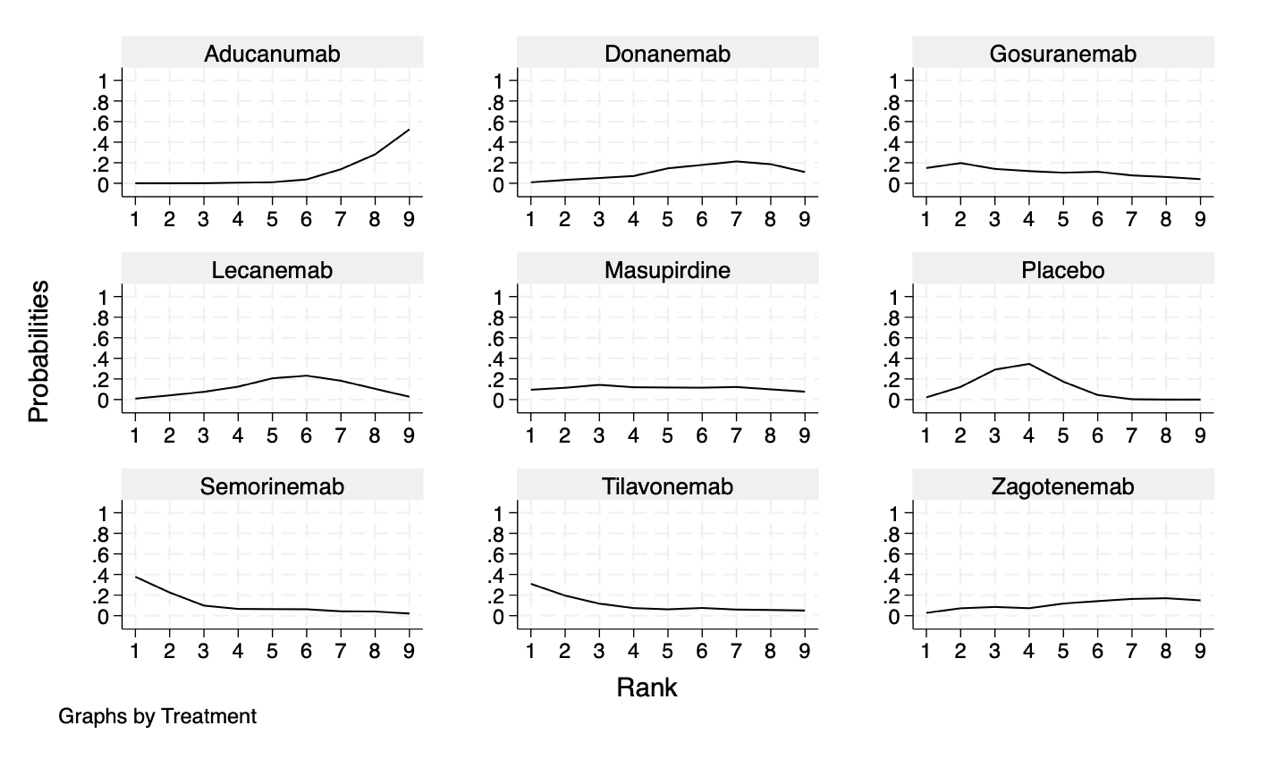
**

**Supplemental Figure 1.** Ranking probability distribution of treatments for CDR-SB outcomes.

| Rank | T1 | T2 | T3 | T4 | T5 | T6 | T7 | T8 | T9 |
| --- | --- | --- | --- | --- | --- | --- | --- | --- | --- |
| Best | 2.6 | 0.0 | 0.9 | 1.1 | 15.7 | 37.5 | 28.7 | 3.6 | 9.9 |
| 2nd | 12.9 | 0.0 | 3.7 | 3.3 | 18.8 | 20.3 | 19.8 | 7.0 | 14.4 |
| 3rd | 29.6 | 0.1 | 7.4 | 5.9 | 14.4 | 10.5 | 11.9 | 8.0 | 12.3 |
| 4th | 32.1 | 0.6 | 12.6 | 8.0 | 10.7 | 7.3 | 8.4 | 9.3 | 11.1 |
| 5th | 17.2 | 1.7 | 20.3 | 13.3 | 10.6 | 6.9 | 7.5 | 11.0 | 11.5 |
| 6th | 5.0 | 4.8 | 23.4 | 18.0 | 10.3 | 6.3 | 7.2 | 13.3 | 11.8 |
| 7th | 0.7 | 12.2 | 18.9 | 20.5 | 9.0 | 4.7 | 6.5 | 15.9 | 11.6 |
| 8th | 0.0 | 28.0 | 10.4 | 19.4 | 6.7 | 3.9 | 5.6 | 16.6 | 9.4 |
| Worst | 0.0 | 52.6 | 2.3 | 10.5 | 4.0 | 2.8 | 4.6 | 15.2 | 8.1 |

**Supplemental Table 1.** Ranking probability table for CDR-SB outcomes.

| Placebo |  | | | | | | | | | |
| --- | --- | --- | --- | --- | --- | --- | --- | --- | --- | --- |
| 0.20 (-1.11,1.51) | Masupirdine |  | | | | | | | |  |
| 0.50 (-0.72,1.72) | 0.30 (-1.50,2.10) | Zagotenemab |  | | | | | | |  |
| -0.22 (-1.82,1.38) | -0.42 (-2.49,1.65) | -0.72 (-2.73,1.29) | Tilavonemab |  |  |  |  |  |  |  |
| -0.41 (-1.99,1.17) | -0.61 (-2.66,1.44) | -0.91 (-2.91,1.09) | -0.19 (-2.44,2.06) | Semorinemab |  | | | | | |
| 0.00 (-1.30,1.30) | -0.20 (-2.05,1.65) | -0.50 (-2.29,1.29) | 0.22 (-1.84,2.28) | 0.41 (-1.63,2.45) | Gosuranemab |  |  | |  |  |
| 0.54 (-0.35,1.43) | 0.34 (-1.25,1.92) | 0.04 (-1.47,1.55) | 0.76 (-1.07,2.59) | 0.95 (-0.86,2.76) | 0.54 (-1.03,2.11) | Donanemab |  |  |  |  |
| 0.36 (-0.29,1.00) | 0.16 (-1.31,1.62) | -0.14 (-1.53,1.24) | 0.58 (-1.15,2.30) | 0.77 (-0.94,2.47) | 0.36 (-1.09,1.81) | -0.18 (-1.28,0.91) | Lecanemab |  |  |  |
| 1.06 (0.42,1.70) | 0.86 (-0.60,2.32) | 0.56 (-0.82,1.94) | 1.28 (-0.44,3.00) | 1.47 (-0.23,3.17) | 1.06 (-0.39,2.51) | 0.52 (-0.57,1.62) | 0.70 (-0.20,1.61) | Aducanumab |  |  |

**Supplemental Table 2.** Forest plot of pairwise comparisons for CDR-SB outcomes (mean difference and 95% CI).

| compname | intercept | se_intercept | t_intercept | p_intercept |
| --- | --- | --- | --- | --- |
| A versus C | -0.8228503 | 0.1991414 | -4.13199 | 0.0538808 |
| A versus B | 16.04923 | 4.356028 | 3.684374 | 0.0664124 |
| A versus D | 2.911172 | 0 | . | . |

**Supplemental Table 3.** **Egger's test for small-study effects for CDR-SB outcomes.** The table presents the results of Egger's regression test, with the intercept, standard error (SE), t-statistic, and p-value. A significant p-value (<0.05) suggests the presence of funnel plot asymmetry, which may indicate publication bias or small-study effects.


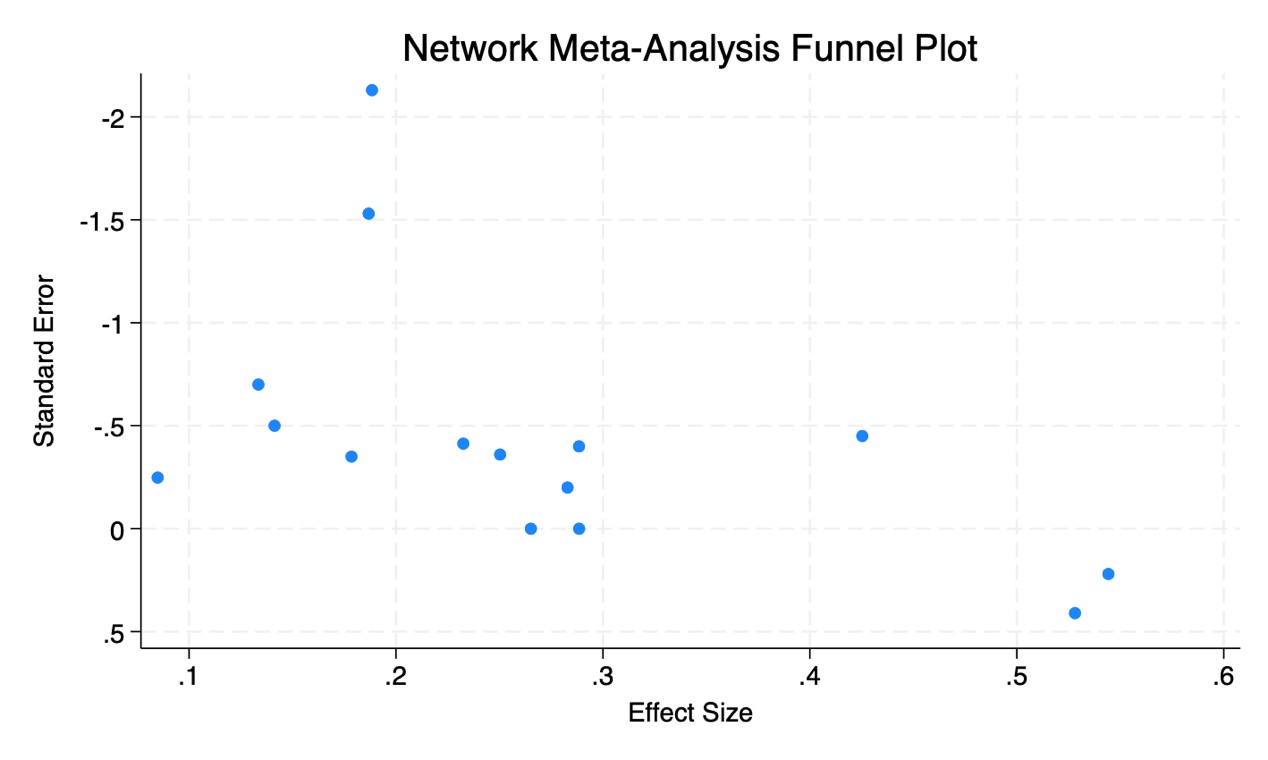


**Supplemental Figure 2.** Funnel plot for publication bias (CDR-SB)

**ADAS-cog**


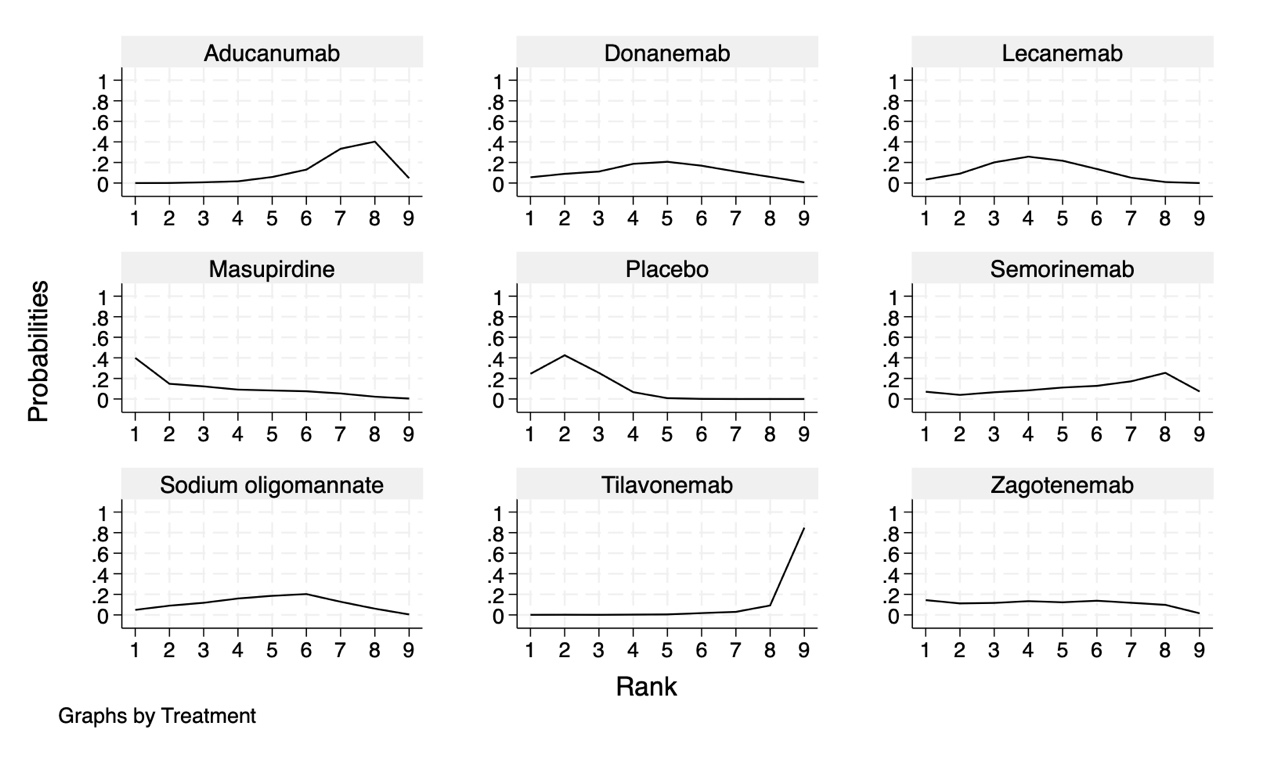


**Supplemental Figure 3.** Ranking probability distribution of treatments for ADAS-cog outcomes

| **Rank** | **T1** | **T2** | **T3** | **T4** | **T6** | **T7** | **T8** | **T9** | **T10** |
| --- | --- | --- | --- | --- | --- | --- | --- | --- | --- |
| **Best** | 25.6 | 0 | 4.2 | 5.3 | 5.3 | 0 | 14.8 | 37.6 | 7.2 |
| **2nd** | 42.4 | 0.2 | 10.1 | 10 | 5.1 | 0.2 | 10.2 | 14.2 | 7.6 |
| **3rd** | 24 | 0.4 | 19.7 | 13.9 | 6.3 | 0 | 10.3 | 12.9 | 12.5 |
| **4th** | 7.3 | 1.3 | 27.1 | 16.2 | 9.6 | 0.3 | 14.2 | 10.1 | 13.9 |
| **5th** | 0.7 | 5.8 | 20.1 | 20.9 | 10.1 | 0.4 | 14.1 | 9.3 | 18.6 |
| **6th** | 0 | 17.6 | 12.6 | 17.3 | 12.5 | 1.2 | 13 | 6.6 | 19.2 |
| **7th** | 0 | 31.3 | 4.9 | 11.6 | 16.1 | 2.9 | 11.9 | 6.3 | 15 |
| **8th** | 0 | 39.9 | 1.3 | 4.2 | 28.5 | 8.2 | 9.3 | 3 | 5.6 |
| **Worst** | 0 | 3.5 | 0 | 0.6 | 6.5 | 86.8 | 2.2 | 0 | 0.4 |

**Supplemental Table 4.** Ranking probability table for ADAS-cog outcomes

| Placebo |  |  |  |  |  |  |  |  |
| --- | --- | --- | --- | --- | --- | --- | --- | --- |
| 1.66 (-1.11,4.44) | Sodium oligomannate |  |  |  |  |  |  |  |
| 0.10 (-3.74,3.94) | -1.56 (-6.30,3.17) | Masupirdine |  |  |  |  |  |  |
| 1.50 (-2.34,5.34) | -0.16 (-4.90,4.57) | 1.40 (-4.03,6.83) | Zagotenemab |  |  |  |  |  |
| 7.40 (2.66,12.14) | 5.74 (0.25,11.23) | 7.30 (1.20,13.40) | 5.90 (-0.20,12.00) | Tilavonemab |  |  |  |  |
| 2.76 (-1.68,7.20) | 1.10 (-4.14,6.33) | 2.66 (-3.21,8.53) | 1.26 (-4.61,7.13) | -4.64 (-11.13,1.85) | Semorinemab |  |  |  |
| 1.58 (-1.13,4.30) | -0.08 (-3.96,3.80) | 1.48 (-3.22,6.19) | 0.08 (-4.62,4.78) | -5.82 (-11.28,-0.36) | -1.18 (-6.38,4.03) | Donanemab |  |  |
| 1.24 (-0.61,3.09) | -0.43 (-3.76,2.91) | 1.14 (-3.13,5.40) | -0.26 (-4.52,4.00) | -6.16 (-11.25,-1.08) | -1.52 (-6.33,3.29) | -0.34 (-3.63,2.94) | Lecanemab |  |
| 3.51 (1.63,5.39) | 1.85 (-1.51,5.20) | 3.41 (-0.87,7.69) | 2.01 (-2.26,6.28) | -3.89 (-8.99,1.21) | 0.75 (-4.07,5.57) | 1.93 (-1.38,5.23) | 2.27 (-0.37,4.91) | Aducanumab |

**Supplemental Table 5.** Forest plot of pairwise comparisons for ADAS-cog outcomes (mean difference and 95% CI).


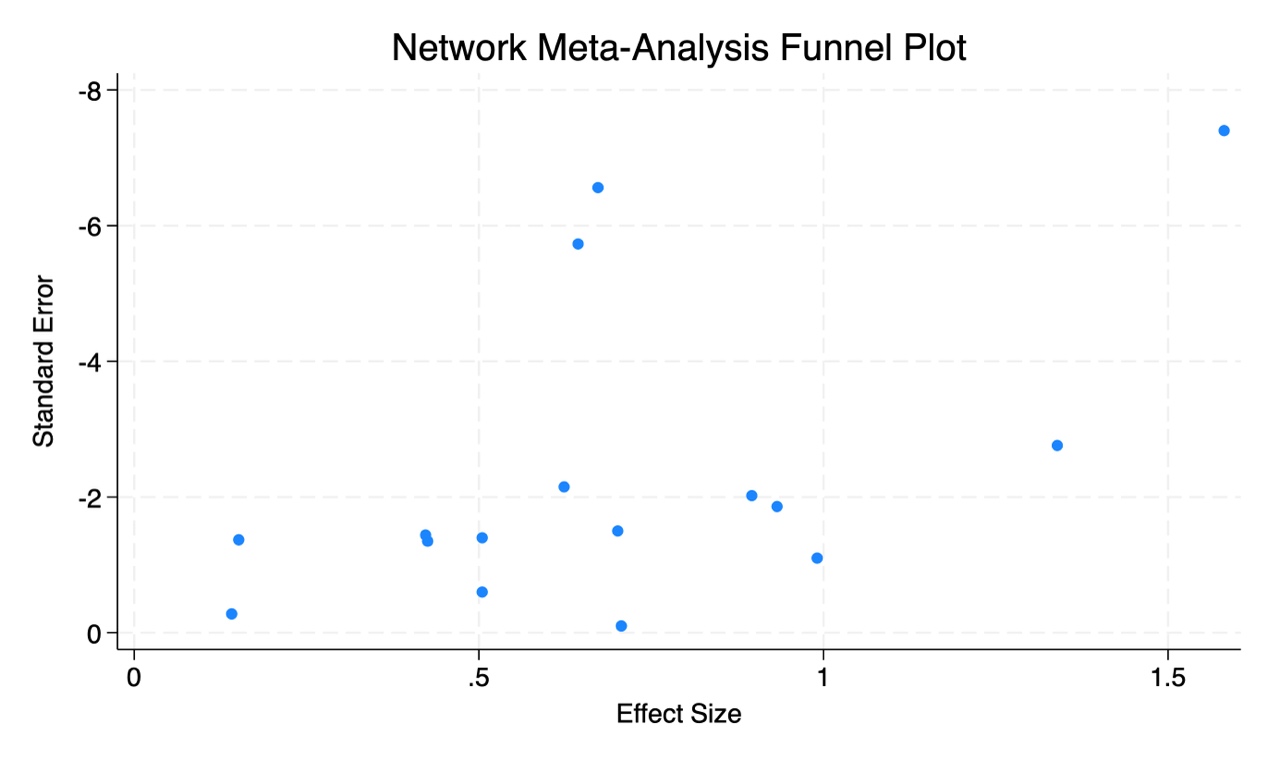


**Supplemental Figure 4.** Funnel plot for publication bias (ADAS-cog).

| compname | intercept | se_intercept | t_intercept | p_intercept |
| --- | --- | --- | --- | --- |
| A versus B | -33.46012 | 3.03755 | -11.0155 | 0.0081407 |
| A versus C | -2.147645 | 3.541992 | -0.606338 | 0.6059453 |
| A versus D | -1.006107 | 0 | . | . |

**Supplemental Table 6.** Node-splitting inconsistency test results for ADAS-cog outcomes.

**MMSE**

**
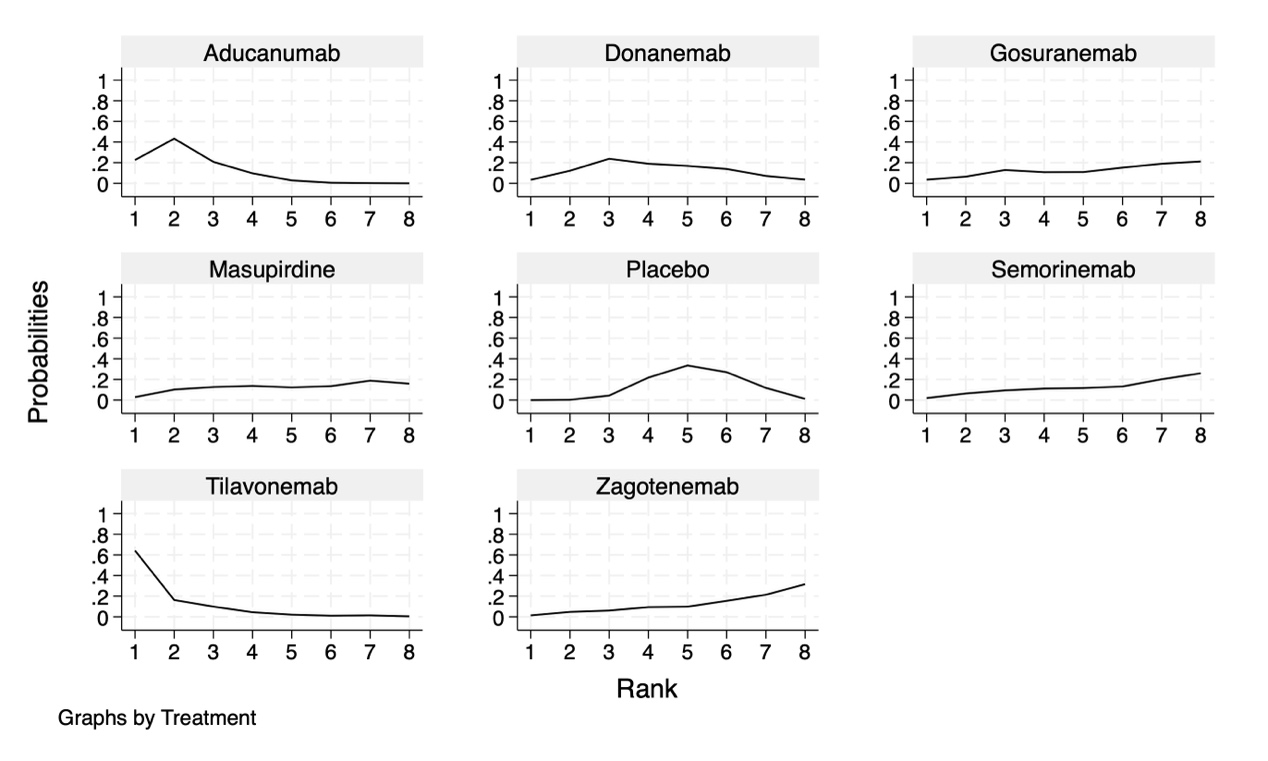
**

**Supplemental Figure 5.** Ranking probability distribution of treatments for MMSE outcomes.

| **Rank** | **T1** | **T2** | **T4** | **T5** | **T6** | **T7** | **T8** | **T9** |
| --- | --- | --- | --- | --- | --- | --- | --- | --- |
| **Best** | 0 | 20.2 | 3.9 | 3.2 | 1.8 | 66.2 | 1.6 | 3.1 |
| **2nd** | 0.2 | 46.5 | 12.3 | 6.2 | 6.5 | 16.1 | 3 | 9.2 |
| **3rd** | 5.5 | 22.2 | 23.2 | 12.6 | 9 | 8.8 | 6.3 | 12.4 |
| **4th** | 20.6 | 7.2 | 21.4 | 12.4 | 10.9 | 4.4 | 9.3 | 13.8 |
| **5th** | 36.6 | 2.8 | 15.1 | 11.5 | 10.6 | 2 | 9.6 | 11.8 |
| **6th** | 27.1 | 1.1 | 13.9 | 13.1 | 15.9 | 1.2 | 12.6 | 15.1 |
| **7th** | 8.5 | 0 | 6.8 | 21.8 | 21.6 | 0.9 | 22.4 | 18 |
| **Worst** | 1.5 | 0 | 3.4 | 19.2 | 23.7 | 0.4 | 35.2 | 16.6 |

**Supplemental Table 7.** Ranking probability table for MMSE outcomes.

| Placebo |  | | |  |  |  |  |
| --- | --- | --- | --- | --- | --- | --- | --- |
| 0.10 (-3.28,3.48) | Masupirdine |  | |  |  |  |  |
| 1.00 (-2.32,4.32) | 0.90 (-3.84,5.64) | Zagotenemab |  |  |  |  |  |
| -3.20 (-6.88,0.48) | -3.30 (-8.30,1.70) | -4.20 (-9.16,0.76) | Tilavonemab |  |  |  |  |
| 0.68 (-2.81,4.17) | 0.58 (-4.28,5.44) | -0.32 (-5.14,4.50) | 3.88 (-1.19,8.95) | Semorinemab |  |  |  |
| 0.34 (-3.07,3.75) | 0.24 (-4.56,5.04) | -0.66 (-5.42,4.10) | 3.54 (-1.48,8.56) | -0.34 (-5.22,4.54) | Gosuranemab |  |  |
| -0.55 (-2.93,1.84) | -0.65 (-4.78,3.49) | -1.55 (-5.64,2.54) | 2.65 (-1.74,7.04) | -1.23 (-5.45,3.00) | -0.89 (-5.05,3.28) | Donanemab |  |
| -1.99 (-3.68,-0.30) | -2.09 (-5.87,1.69) | -2.99 (-6.72,0.74) | 1.21 (-2.84,5.27) | -2.67 (-6.54,1.21) | -2.33 (-6.14,1.48) | -1.44 (-4.37,1.48) | Aducanumab |

**Supplemental Table 8.** Forest plot of pairwise comparisons for MMSE outcomes (mean difference and 95% CI).


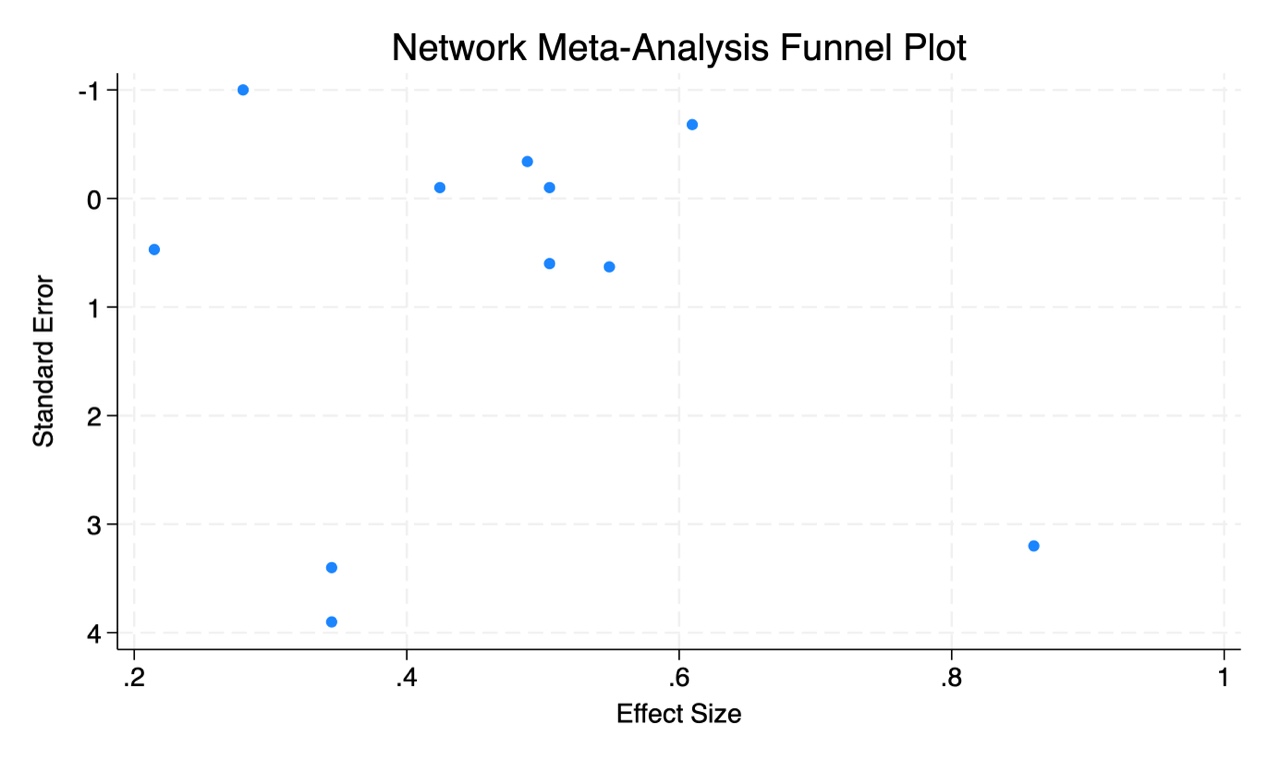


**Supplemental Figure 6.** Funnel plot for publication bias (MMSE)

| compname | intercept | se_intercept | t_intercept | p_intercept |
| --- | --- | --- | --- | --- |
| A versus B | -21.25586 | 2.71288 | -7.835165 | 0.0159018 |
| A versus C | 0.4790473 | 0 | . | . |

**Supplemental Table 9.** Egger's test for small-study effects for MMSE outcomes.
